# Endovascular repair of type B acute aortic syndromes involving the left subclavian artery: A retrospective single-centre study

**DOI:** 10.1371/journal.pone.0328817

**Published:** 2025-08-01

**Authors:** Mengyang Kang, Yang Zhao, Yan Meng, Qiang Ma, Junbo Zhang, Hao Qin, Hongyan Tian

**Affiliations:** Department of Peripheral Vascular Diseases, The First Affiliated Hospital of Xi’an Jiaotong University, Shaanxi, China; Royal Holloway University of London, UNITED KINGDOM OF GREAT BRITAIN AND NORTHERN IRELAND

## Abstract

**Background:**

The mid-term outcomes of using the Castor single branched stent graft to treat patients with type B acute aortic syndromes (AASs) involving the left subclavian artery (LSA) were unclear. This study aims to evaluate the mid-term efficacy of the Castor single branched stent graft in the treatment of type B AASs involving the LSA.

**Methods:**

The retrospective study enrolled patients with type B AASs involving the LSA who were consecutively admitted to our hospital between January 1, 2019 and October 30, 2023. Patients underwent thoracic endovascular aortic repair (TEVAR) using the Castor device. The clinical data of these patients were collected, and their outcomes were followed.

**Results:**

This study included 106 patients, comprising those presenting with aortic dissection (AD) (n = 93), penetrating aortic ulcer (PAU) (n = 9), and intramural haematoma (IMH) (n = 4). All patients achieved the technical success. After a median follow-up period of 454 days (range: 359–771 days), all patients demonstrated survival, with 14 (14.4%) patients experiencing complications, including acute myocardial infarction (AMI, n = 1), endoleaks (n = 7), stenosis of the branched section(n = 3), stent graft-induced new entry tear (SINE, n = 2), and progressive aortic dilation (n = 1). Among the endoleaks, there were 1 Type I_b_, 2 Type II, and 4 Type IV cases. Among the enrolled patients, two required re-intervention. No retrograde type A aortic dissection (RTAD), paraplegia or stroke were observed during the follow-up period.

**Conclusion:**

The Castor device provided an easily manipulated, safe, and effective treatment option for patients with type B AASs involving the LSA.

## Introduction

Acute aortic syndromes (AASs), including aortic dissection (AD), intramural haematoma (IMH), and penetrating aortic ulcer (PAU), necessitates immediate evaluation and treatment [1]. The various AASs subtyped result from the extent of rupture in the aortic wall that can range from localized and isolated medial disruption in IMH to widespread and diffuse medial damage in classical AD. Over the past decade, thoracic endovascular aortic repair (TEVAR) has emerged as the primary treatment approach for type B AASs and has replaced open surgical repair [2].

In the past, coverage of the left subclavian artery (LSA) was necessary to create an adequate proximal landing zone (PLZ) for managing patients with type B AASs involving LSA [3]. However, studies have demonstrated that covering the LSA can lead to complications such as limb ischaemia, paraplegia, and acute cerebral infarction due to compromised blood supply [4]. Therefore, the updated guidelines have recommended reconstructing the LSA for patients with aortic lesions involving it [5]. The current revascularization techniques employed for the LSA include extra-anatomic bypass, parallel stent grafts, and fenestrated or branched endograft. However, there are certain limitations in their clinical management. An extra-anatomic bypass entails substantial surgical trauma, parallel stent deployment carries inherent risks of postoperative endoleaks, while the fenestration technique is intricate and may pose potential legal risks.

Castor single branched stent graft (Microport Endovascular Medtech [Group] Co. Ltd, Shanghai, China) was a unibody endograft with a branched section, which enable exclude the primary entry tear and revascularize the LSA. To address the above challenges, the Castor device appears to be the most optimal approach as it aligns well with the natural morphology of the aorta and hemodynamic features [6]. Nevertheless, the clinical outcomes of using the Castor single branched stent graft to treat patients with type B AASs involving the LSA remain unclear. Therefore, this retrospective study aimed to evaluate the mid-term outcomes of the Castor device for treating type B AASs involving the LSA.

## Patients and methods

This retrospective study was approved by the Ethics Committee of the First Affiliated Hospital of Xi’an Jiaotong University. The requirement for individual informed consent is waived, because of the retrospective nature of the study. (No: XJTU1AF2022LSK-234).

### Study design and patients

**Fig 1:**
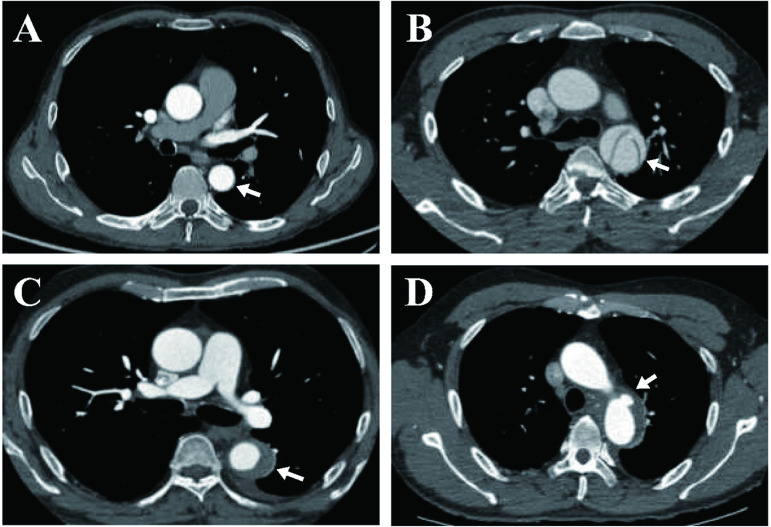
Representative CT angiography images of different types of acute aortic syndromes are shown. **(A)** Normal aorta; **(B)** Aortic dissection; **(C)** intramural hematoma; **(D)** Penetrating aortic ulcer. The arrows indicate the aorta.

Our medical centre performed TEVAR with the Castor device on a total of 106 patients between January 1, 2019 and October 30, 2023, who had been diagnosed with type B AASs involving the LSA by computed tomography angiography (CTA) or digital subtraction angiography (DSA). The patients with aortic arch anomalies were excluded, including aberrant right or left subclavian artery, isolated left vertebral artery, and right-side of aortic arch, etc. We anonymized the patients’ personal information and retrospectively collected data on them, including demographic characteristics, clinical manifestations, aortic morphological parameters, operative procedure details, and mid-term outcomes. Aortic morphological parameters were conducted based on the preoperative CTA images (**Fig 1**).

### The Castor single branched stent graft

**Fig 2:**
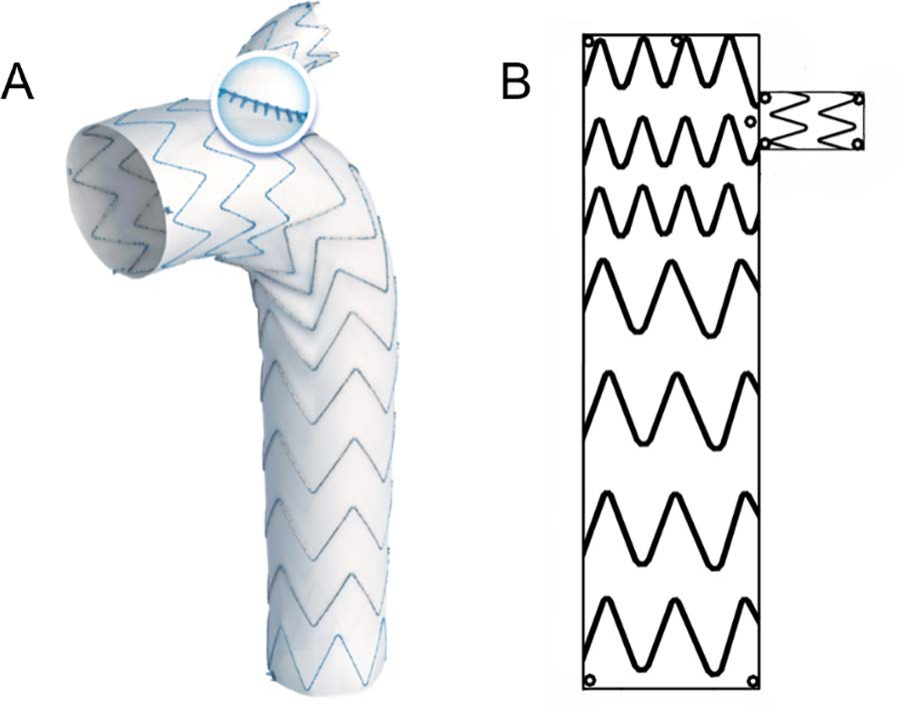
The physical and schematic diagram of the Castor single branched stent graft.

The Castor device was made of nitinol and polyester and was composed of a main trunk and the side branched section, which was specifically designed to exclude the primary entry tear while preserving the blood supply of the LSA. The physical and schematic diagram is shown in **Fig 2**.

In addition to completely excluding the proximal entry tear, the prevention of aortic wall injury, reduction in postoperative complications, and achievement of robust long-term outcomes are also desirable therapeutic goals. Hence, the delivery system, the structure of the stent graft, and the details of operative procedure were designed to attain the treatment goals. (i) A smaller diameter delivery system can avoid access site complications. (ii) The outer sheath does not enter the aortic arch but remains in the descending aorta during the procedure. When the orientation of the first introduction is inaccurate, the stent graft can be retrieved from the aortic arch, back into the outer sheath, and then rotated to the correct orientation. In this way, manipulation in the aortic arch can be reduced, to reduce the risk of intimal injury and cerebral embolization. (iii) The soft inner sheath wrapped around the stent graft, made with soft polyester fabric, was the second protection for the aortic intima. (iv) The junction between the branched section and the main trunk of the Castor device is made of polyester fabric, allowing the branched section to be easily drawn into the LSA, and conform to the morphology of the LSA. (v) The Castor device is offered in various lengths and diameters, and the branched section originates at varying distances (ranging from 5 to 30 mm) from the proximal edge of the main trunk. It can be customized according to the aortic anatomy of patients.

### Endovascular procedure

The patients were administered optimal medication to manage blood pressure (systolic blood pressure within range of 100–120 mmHg) and heart rate (60–80 beats per minute) preoperatively. The final size of the Castor single branched stent graft was determined by the combined measurements obtained from preoperative CTA and intraoperative DSA. To prevent thrombosis during the TEVAR, systemic heparinization is achieved by administering unfractionated heparin via intravenous injection at an initial dose of 80 IU/kg.

**Fig 3:**
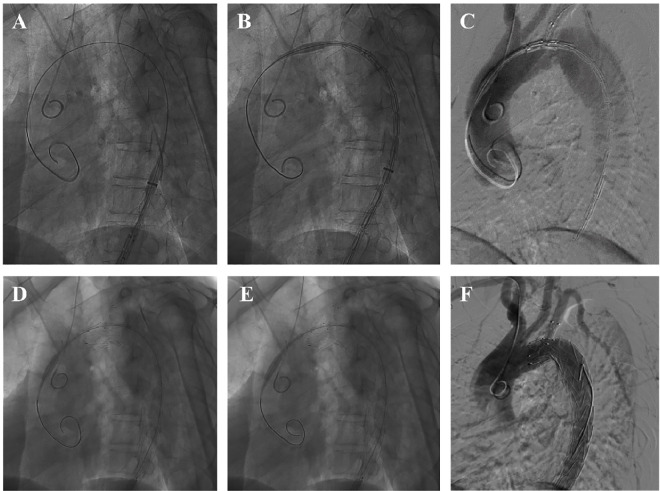
Operative procedure. Representative DSA images acquired from one patient are shown. **(A)** The introduction of the delivery system and guiding wire. **(B)** The insertion of the Castor device to the aortic arch. **(C)** The positioning the Castor device at the predetermined location through angiography. **(D)** The expansion of the main trunk of the Castor device. **(E)** The expansion of the branch section of the Castor device. **(F)** Intraoperative aortogram showing the robust postoperative outcome.

A catheter was advanced into the brachiocephalic artery via the right radial artery puncture and positioned in the ascending aorta segment, primarily for assisting angiographic localization and measuring the pressure inside the aortic cavity. An arterial puncture was performed on one side of the femoral artery to introduce the delivery system. A Mark pigtail catheter and guidewire were threaded from the femoral artery to the ascending aorta through the true lumen, followed by exchange for a super stiff guidewire (Lunderquist; Cook, Bloomington, IN, US). The Castor device was inserted into the descending aorta via the super stiff guidewire, while the traction wire of the branched section was pulled from the left brachial artery. As the stent graft was guided up to the level of the LSA, it was crucial to ensure that the branched section of the Castor device followed the greater curvature of the aortic arch without any wrapping by the traction wire. Otherwise, the Castor device has the potential to be retracted into the outer sheath positioned in the descending aorta, rotated for achieving proper alignment, and subsequently reintroduced into the aortic arch. In cases where the branched section follows the greater curvature of the aortic arch, both traction wires of the branched section and main trunk delivery systems run parallel while simultaneously removing the soft inner sheath and pulling the branched section into the LSA. The stent graft is then precisely positioned as planned, followed by intraoperative angiography. Finally, an expansion of both the main trunk and branched section of the Castor device is carried out (**Fig 3**).

The exclusion of the primary entry tear and patency of the stent graft were evaluated through immediate aortograms. The delivery system and guidewires were removed. As part of the postoperative regimen, antiplatelet therapy with aspirin (100 mg/day) or clopidogrel (75 mg/day) for was implemented as a preventive measure against thrombosis in the branched section of the Castor device.

### Endpoints and follow-up

The primary outcome was the technical success rate and mortality. The secondary outcomes were complications and the re-intervention rate. Complications including aortic rupture, retrograde type A aortic dissection (RTAD), stent graft-induced new entry tear (SINE), endoleaks, acute myocardial infarction (AMI), stroke, paraplegia/paraparesis, and stenosis of the branched section. Mid-term outcomes were documented through outpatient clinic visits or telephone interviews. Standardised imaging follow-up was performed employing CTA at intervals of 1, 3, and 6 months postoperatively and annually postoperation thereafter.

### Definitions

Technical success was defined as the successful deployment of the Castor stent at the predetermined location, with exclusion the primary entry tear and reconstructed the LSA. The delivery system was fully withdrawn from the body without complications, and no type I or type III endoleaks were observed. The duration of the operation is defined as the time from the beginning of femoral artery puncture to the successful release of the Castor device confirmed by angiography. The hyperacute phase was defined as the period within 1 day after disease onset. The acute phase was 1–14 days. The subacute phase was 15–90 days. The chronic phase was exceeding 90 days.

### Statistical analysis

The normality of continuous variables was assessed using the Shapiro-Wilk test. Mean ± standard deviation was used to present continuous variables with a normal distribution, while skewed variables were described by median and interquartile range. Categorical variables were presented as frequency and percentage. Data analysis was conducted using IBM SPSS Statistics 26.0 software (Chicago, IL, USA), and GraphPad Prism 9.0 software was utilized to visualize the cumulative probability curve of complications.

## Results

### Baseline clinical features

Between January 1, 2019 and October 30, 2023, a consecutive series of 106 patients (88 men, mean age 53.2 ± 13.5 years) presenting with type B AASs involving the LSA underwent TEVAR using the Castor device and included cases involving AD (87.7%, n = 93), PAU (8.5%, n = 9), and IMH (3.8%, n = 4). 68 (60.2%) patients had the history of hypertension, among them, only one-third of patients received regular medical treatment, and a mere 17.6% achieved optimal blood pressure levels. The baseline characteristics are shown in **Table 1**.

**Table 1:** Baseline characteristics.

Variables	Type B AASs (n = 106)
Age, years	53.2 ± 13.5
Men	88 (83.0)
BMI, kg/m²	25.5 ± 3.5
Classifications	
AD	93 (87.7)
PAU	9 (8.5)
IMH	4 (3.8)
Comorbidity	
Hypertension (n = 68)	68 (64.2)
Regular medical treatment	22 (32.4)
Optimal blood pressure controlled	12 (17.6)
CAD	11 (10.4)
Previous stroke	5 (4.7)
Diabetes	5 (4.7)
Tumour	3 (2.8)
Smoking	49 (46.2)
Drinking	10 (9.4)

Values are given as mean ± standard deviation or n (%). AASs, acute aortic syndromes; AD, aortic dissection; BMI, body mass index; CAD, coronary artery disease; IMH, intramural haematoma; PAU, penetrating aortic ulcer.

The most common symptom was sudden chest pain (57.5%, n = 61). The mean systolic blood pressure (SBP), diastolic blood pressure (DBP), and heart rate at admission were 153.3 ± 21.8 mmHg, 91.6 ± 15.1 mmHg, and 83.6 ± 11.1 beats/min, respectively. The indications for acute TEVAR included refractory pain (26.4%, n = 28), uncontrolled hypertension (8.5%, n = 9), aortic rupture (0.9%, n = 1), and malperfusion syndrome (5.7%, n = 6). The clinical manifestations are shown in Table 2.

**Table 2:** Clinical manifestations.

Variables	Type B AASs (n = 106)
Sudden chest pain	61 (57.5)
SBP, mmHg	153.3 ± 21.8
DBP, mmHg	91.6 ± 15.1
Heart rate, beats/min	83.6 ± 11.1
Acute complication	
Refractory pain	28 (26.4)
Uncontrolled hypertension	9 (8.5)
Aortic rupture	1 (0.9)
Malperfusion syndrome	6 (5.7)
Mesenteric malperfusion	0
Extremity malperfusion	3 (2.8)
Renal malperfusion	2 (1.9)
Cerebral malperfusion	0
Cardiac malperfusion	1 (0.9)
Spinal malperfusion	0
Pericardial effusion	4 (3.8)
Pleural effusion	11 (10.4)

Values are given as mean ± standard deviation or n (%). AASs, acute aortic syndromes; DBP, diastolic blood pressure; SBP, systolic blood pressure; DBP, diastolic blood pressure.

### Morphological characteristics and endovascular management

Ten patients underwent emergency endovascular repair due to clinical conditions, without preoperative CTA assessment. The morphological parameters of the aortic assessment for the remaining 96 patients were shown in **Table 3**. Accurate preoperative assessment is crucial for the selection of Castor device size. Specific endograft parameters are detailed in **Table 3**. 67.8% of the patients (n = 70) underwent TEVAR during the acute phase. The mean procedural time was 46.5 minutes (range: 34.0–60.3 minutes). During the delivery of the Castor device to its designated position, the main trunk and the guidewire of the branch section of 17 patients (16%) were entangled. To address this issue, the stent graft was retracted into the outer sheath positioned in the descending aorta, followed by rotating the delivery handle to resolve the entanglement. Subsequently, the covered stent was advanced to the aortic arch and precisely deployed at the predetermined position. Importantly, after these adjustments were made, all stent grafts were deployed successfully at the intended landing zone, with no cases of device failure, migration, or deployment abortion. Balloon dilation was performed on the branched section of the Castor device in eight (7.5%) patients due to torsion or stenosis. The final intraoperative angiography revealed an immediate type I endoleak that was successfully resolved through balloon dilation.

**Table 3:** Morphological characteristics and endovascular management of the aorta.

Variables	Type B AASs (n = 96)
Aortic measurements **(n = 96)**	
The mean aortic diameter of the distal part of the LCCA, mm	30.3 ± 2.9
The mean aortic diameter of the distal part of the LSA, mm	29.3 ± 2.7
The mean aortic diameter of the distal part of the descending aorta, mm	23.2 ± 3.4
The mean diameter of the distal part of the LSA, mm	9.8 ± 1.7
The median distance of the LCCA-to-LSA, mm	11.2 ± 5.6
The number of aortic branch vessels involved (excluding the LSA)	
1	10 (9.4)
2	3 (2.8)
3	5 (4.1)
4	3 (2.8)
5	1 (0.9)
Specification parameters of the Castor devices	
The mean diameter of the proximal end of the main trunk, mm	31.9 ± 2.59
The mean diameter of the distal end of the main trunk, mm	25.9 ± 2.6
The mean diameter of the distal end of the branch section, mm	10.9 ± 1.0
The length of the main trunk, mm	200
The length of the branch section, mm	26.0 ± 2.0
The Length from branch section to the proximal end of main trunk	10 (5- 15)
Operative procedure **(n = 106)**	
The duration of the operation	46.5 (34.0-60.3)
The volume of contrast agent, ml	190 (170-200)
Postoperative length of hospital stays, day	4 (3-5)
Time of operation	
Hyperacute (<24 hours)	3 (2.8)
Acute (1–14 days)	70 (66.0)
Subacute (15–90 days)	31 (29.2)
Chronic (>90 days)	2 (1.9)
Relieve the winding of the delivery system to adjusted in direction	
No	89 (84.0)
Yes	17(16.0)
The number of endografts	
1	87 (82.1)
2	19 (17.9)
Balloon dilation of the branched section	8 (7.5)
Intraoperative endoleaks	1 (0.9)

Values are given as mean ± standard deviation, median and interquartile range, or n (%). AASs, acute aortic syndromes; LCCA, left common carotid artery; LSA, left subclavian artery.

### In-hospital outcomes

All patients had achieved the technical success (100%). One patient died during the hospitalization due to acute anterior inferior myocardial infarction. Five patients experienced complications, including: three cases of pseudoaneurysm at the puncture site of the femoral artery, which were successfully resolved through ultrasonography-guided thrombin injection; one case of pneumonia that was treated and resolved with antibiotics; and one case of renal failure, likely representing an acute exacerbation of pre-existing chronic kidney disease, potentially triggered by contrast-induced nephropathy. No cases of aortic rupture, RTAD, stroke, or paraplegia were observed in this study (**Table 4**).

**Table 4:** In-hospital outcomes.

Variables	Type B AASs (n = 106)
Primary outcomes	
Mortality	1 (0.9)
Secondary outcomes	
Complications	5 (4.6)
Aortic rupture	NA
RTAD	NA
Paraplegia/paraparesis	NA
Stroke	NA
SINE	NA
AMI	NA
Access site complication	3 (2.8)
Pneumonia	1 (0.9)
Renal failure	1 (0.9)

Values are given as mean ± standard deviation, median and interquartile range, or n (%). AASs, acute aortic syndromes; AMI, acute myocardial infarction; RTAD, retrograde type A aortic dissection; SINE, stent graft-induced new entry tear.

### Mid-term outcomes

**Fig 4:**
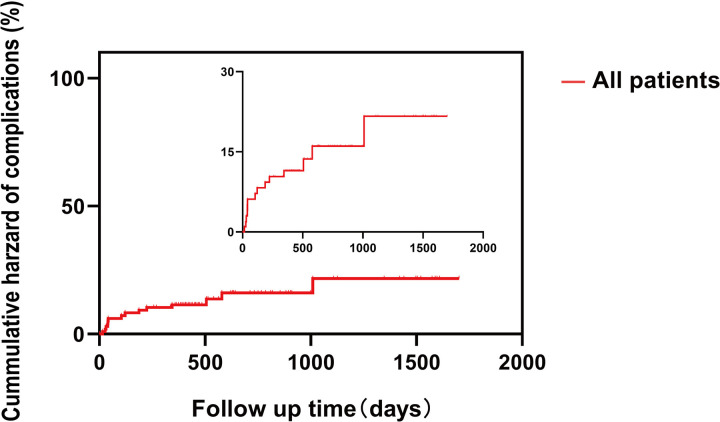
Cumulative probability curves of complications in the mid-term outcome.

After a median follow-up period of 454 days (range: 359–771 days), the rate of loss to follow-up was 8.5% (n = 9). No mortality was observed. Fourteen (14.4%) patients experienced complications that included cases of Acute myocardial infarction (n = 1), SINE (n = 2), endoleaks (n = 7), progressive aortic dilation (n = 1), and stenosis of the branch section (n = 3). The cumulative probability curve of complications is shown in **Fig 4**. Three patients underwent re-intervention. The first patients received PCI for AMI. The second patient was observed to have a SINE 32 days after the procedure, with a distal covered stent implanted. The third patient exhibited stenosis of the branched section during imaging follow-up 19 months after the procedure and was effectively managed through balloon dilation.

Additionally, two other patients exhibited stenosis of the branched section characterized by right vertebral artery dominance and an absence of significant clinical symptoms, while another patient with SINE declined further re-intervention. Therefore, meticulous monitoring is needed. Meanwhile, seven cases of endoleak were observed, including Type I_b_ endoleak (n = 1), Type II (n = 2) and Type IV endoleak (n = 4). We implanted a covered stent at the distal end of main trunk of Castor device for the patient with Type I_b_ endoleak. Type II endoleaks in these patients were caused by intercostal arteries. Since the endoleaks were minor, close surveillance was adopted. Type IV endoleaks predominantly occur in patients with large primary entry tears and typically resolve within a short period postoperatively. In 97 patients with type B AASs, the last CTA examination demonstrated complete thrombosis in the false lumen occurred in 78 (80.4%) patients (**Table 5**).

**Table 5:** Mid-term outcomes.

Variables	Type B AASs (n = 97)
The median follow-up time, day	454 (359-771)
Primary outcomes	
Mortality	0 (0)
Secondary outcomes	
Complications	14 (14.4)
SINE	2 (2.1)
Endoleak	7 (7.2)
Type IV endoleak	4 (4.1)
Type I_b_ endoleak	1 (1.0)
Type II endoleak	2 (2.1)
AMI	1 (1.0)
Stenosis of branched section	3 (3.1)
Progressive aortic dilatation	1 (1.0)
Re-intervention	3 (3.1)

Values are given as mean ± standard deviation, median and interquartile range, or n (%). AASs, acute aortic syndromes; AMI, Acute myocardial infarction; SINE, stent graft-induced new entry tears.

## Discussion

The main findings of the study can be summarized as follows. First, the overall technical success rate was 100%, and no patients converted to open surgical repair. Second, the use of Castor device mitigated the risk of complications. Third, the Castor device performed well in the management of patients with type B AASs involving the LSA at the acute phases.

Currently, the techniques of LSA revascularization are as follows. (i) The chimney technique inherently presents a potential risk of postoperative endoleak [7], necessitating additional monitoring to determine its long-term patency rate [8]; (ii) The fenestration can be categorized into *in-situ* or fenestration, both of which compromise the integrity of the endograft and increase the risk of type III endoleak, thereby increasing the risk of stent migration and deformation, and potentially leading to the misalignment of the ostium of branch artery [9]. The *in-situ* fenestration necessitates employing needle, laser, or radiofrequency punctures that involve a complex procedure in the aortic arch, resulting in an elevated risk of neurological complications postoperatively [10]; (iii) The carotid-subclavian artery bypass needs general anaesthesia, implying a greater risk of surgical injury and vascular prosthetic graft infection; (iv) The LSA revascularization with the branched stent graft is in accordance with the structure and function attributes of the aorta, minimizing the risk of postoperative endoleak. Therefore, reconstruction of the LSA with the branched stent graft is a safe and effective approach.

In 1999, Inoue et al. [11] first reported the utilization of one-piece branched stent graft, but it was observed that the branch section caused aortic wall injury upon being pulled into the LSA and was associated with a relatively high rate of complications, including endoleaks, cerebral infarction, and access site complications. However, The Castor single branched stent graft is characterized by its integrated design, allowing the branch segment to rotate multi-directionally up to 150 degrees. This enables precise adaptation to the varying anatomical positions of the LSA opening, while the presence of the endomembrane of the covered stent graft reduces the damage to the vascular wall caused by the stent. This design, on the one hand, avoids hemodynamic disorders caused by the mismatch between the stent graft and vascular anatomy in traditional treatment, ensuring the physiological blood flow reconstruction of the LSA [6]. For patients with type B AASs, precise LSA reconstruction facilitates the restoration of blood supply to the left upper limb and the vertebrobasilar arteries, thereby reducing the risks of stroke, paraplegia, and limb paralysis. One the other hand, traditional LSA reconstruction techniques, such as chimney grafting and fenestration, are associated with a potential risk of endoleak due to their inherent limitations. However, the Castor single branched stent graft, with its unique branch design, significantly reduces the incidence of endoleak. Its excellent collateral patency and close apposition to the aortic wall minimize the impact of blood flow on the gap between the stent graft and the aortic wall, further lowering the risk of endoleak. Secondly, the Castor single branched stent graft, by reconstructing the LSA, extends the proximal landing zone (PLZ) to the aortic arch region (e.g., Ishimaru classification Zone 2). This design with an extended PLZ serves two purposes: (i) it broadens the indications for endovascular therapy, enabling safer minimally invasive treatment options for patients who previously required traditional open surgery or hybrid technique, which reduces the risks of iatrogenic trauma and perioperative complications; (ii) the expanded PLZ, combined with the double-anchoring structure at the proximal end of both the main trunk and the branched section effectively addresses concerns related to stent migration, distortion, and deformation caused by hemodynamic forces.

Recently, many studies have demonstrated the significant efficacy of the Castor device in treating type B aortic dissection (TBAD) involving the LSA. The initial feasibility study (IFS) conducted by Jing et al. [12] have previously demonstrated the safety and efficacy in the managing TBAD. A 5-year postoperative follow-up of 73 patients showed an overall survival rate of 93.2% and a LSA patency rate of 92.6%. Kong et al. [13] endeavored to apply the Castor device in a diverse range of aortic diseases with a limited sample size, yielding favorable outcomes. However, limited research has focused on the mid- or long-outcomes in patients with type B AASs. The study aims to provide clinical evidence in this area. To date, the study represents the largest sample size investigation utilizing Castor device for endovascular treatment of type B AASs.

Accurate preoperative assessment and size selection are key to the technical success of stent deployment. We suggest that preoperative CTA be employed to evaluate the aortic morphological parameters of patients. Additionally, we have improved the TEVAR procedure as follows. (i) We used the super-selective LSA guidewire “drilling” method to draw the guidewires from the LBA that avoids the necessity of using a grabber to capture the branch guidewire, and effectively reduces the operation cost and time; (ii) During delivery of the Castor device, the traction wire of the branched section should remain parallel to the traction wire of the main trunk outside the body of the patient and follow the aorta, reducing winding with the traction wire of the main trunk.

The Castor device can effectively dilate the distal true lumen of the aorta for patients with severe stenosis of the distal edge of the true lumen. The TEVAR procedure with Castor device only requires the one-time introduction and deployment of a branched section. The operative procedure is easy to operate and has a short learning curve, which makes it especially suitable to be carried out in those primary medical centres with less experience of endovascular treatment.

During the postoperative monitoring of our patient cohort, we have identified certain limitations, which were not attributable to the use of the Castor single branched stent graft but rather represented common challenges inherent in TEVAR. For example, we observed that the degree of thrombosis of the false lumen in the distal arch and proximal descending aorta was obvious. However, the extent of thrombosis of the false lumen in the distal descending aorta was low, because we put the distal entry tear aside, and blood flow continued into the false lumen. This is a common problem that TEVAR needs to resolve. To achieve complete aortic remodeling, strict blood pressure control and close observation of the distal entry tear is needed, and prompt re-intervention should be undertaken as soon as needed at the subsequent follow-up. In the future, more attention should be paid to the relevant indicators of aortic remodeling to evaluate the long-term outcome. There were two patients who experienced SINE in this study. We attribute this primarily to the mismatch between the diameter of the distal stent and that of the aorta, leading to excessive oversizing, which consistent with the factors contributing to SINE following TEVAR procedures involving other types of stent grafts. Meanwhile, access site complications were observed in three patients, prompting us to recommend further reducing the size of the delivery system.

Fortunately, the Castor device has been upgraded to Cratos branched aortic stent graft system (MicroPort Endovascular Medtech [Group] Co. Ltd, Shanghai, China). Cratos devices adopts a new release technique that prioritizes deployment of the stent grafts on the inner curvature of the aortic arch and realizes the adjustable function of the proximal end of the stent grafts, which significantly improves the accuracy of stent positioning and effectively eliminates the occurrence of bird beak configurations at the proximal end of the stent grafts, significantly improve the adhesion performance. In addition, the outer sheath diameter of the delivery system has been reduced from 24F to 22F, further reducing the risk of complications at the access site. Through the introduction of innovative techniques such as a longer outer sheath tube, a rotational quick-pull release method, and a release error-proof mechanism, Cratos devices has significantly simplified the endovascular operation. Further research is warranted to assess the performance and clinical outcomes of the upgraded device.

## Conclusion

The Castor device provides a novel, safe, effective, and easily-manipulated endovascular treatment option for patients with type B AASs involving LSA.

## Supporting information

S1 Data The total database of the single-center study on patients with type B acute aortic syndromes. (XLSX)
